# The heterogeneous nucleation of threading dislocations on partial dislocations in III-nitride epilayers

**DOI:** 10.1038/s41598-020-74030-y

**Published:** 2020-10-15

**Authors:** J. Smalc-Koziorοwska, J. Moneta, P. Chatzopoulou, I. G. Vasileiadis, C. Bazioti, Ø. Prytz, I. Belabbas, Ph. Komninou, G. P. Dimitrakopulos

**Affiliations:** 1 Institute of High Pressure Physics, Polish Academy of Sciences, 01-142 Warsaw, Poland; 2 Physics Department, Aristotle University of Thessaloniki, 54124 Thessaloniki, Greece; 3 Department of Physics, Centre for Materials Science and Nanotechnology, University of Oslo, Blindern, 0316 Oslo, Norway; 4 Chemistry Department of Abderahmane, Mira University, 06000 Bejaïa, Algeria

**Keywords:** Semiconductors, Surfaces, interfaces and thin films, Condensed-matter physics, Materials for devices, Electronic devices

## Abstract

III-nitride compound semiconductors are breakthrough materials regarding device applications. However, their heterostructures suffer from very high threading dislocation (TD) densities that impair several aspects of their performance. The physical mechanisms leading to TD nucleation in these materials are still not fully elucidated. An overlooked but apparently important mechanism is their heterogeneous nucleation on domains of basal stacking faults (BSFs). Based on experimental observations by transmission electron microscopy, we present a concise model of this phenomenon occurring in III-nitride alloy heterostructures. Such domains comprise overlapping intrinsic I_1_ BSFs with parallel translation vectors. Overlapping of two BSFs annihilates most of the local elastic strain of their delimiting partial dislocations. What remains combines to yield partial dislocations that are always of screw character. As a result, TD nucleation becomes geometrically necessary, as well as energetically favorable, due to the coexistence of crystallographically equivalent prismatic facets surrounding the BSF domain. The presented model explains all observed BSF domain morphologies, and constitutes a physical mechanism that provides insight regarding dislocation nucleation in wurtzite-structured alloy epilayers.

## Introduction

Edge type threading dislocations (TDs) comprise the great majority of defects in III-nitride epitaxial layers. Such TDs have **a**-type (1/3 < $\overline{1}\overline{1}$20 >) Burgers vectors and appear in very high densities (typically 10^8^–10^10^ cm^-2^)^1^. Their elimination has great technological importance for (opto)electronic device performance, as they affect diverse phenomena like parasitic luminescence^2,3^ segregation^4–6^, electron mobility^7–9^ and device degradation^10,11^^.^ They may be introduced to accommodate small-angle misorientations between initially-formed islands^12^, but also appear in high densities in epilayers deposited in two-dimensional growth mode. The well-known Matthews-Blakeslee mechanism of half-loop dislocation glide to relieve the epitaxial misfit^13^ is not pertinent since, in (0001) III-nitride epilayers, there is no resolved shear stress on the basal or prismatic planes. Activation of pyramidal slip systems like < $\overline{1}\overline{1}$2$\overline{3}$ > {$\overline{1}\overline{1}$22} has been observed, but **a** + **c** dislocations are then generated^14,15^. Overall, the full extent of mechanisms leading to **a**-type TD introduction is still rather unclear.

Recently, it has been recognized that basal stacking faults (BSFs) may be an important source of **a**-type TDs^16–19^ which is a phenomenon that deviates from common perceptions of how TDs are introduced in heteroepitaxy. In the manner that this was first explained^17^, it necessitates the transformation of Shockley partial dislocations (PDs) to **a**-type TDs, which is energetically unfavorable. Shockley PDs with 1/3 < 10$\overline{1}$0 > Burgers vectors delimit the I_2_ intrinsic BSFs, and are usually the result of strain-induced dissociation of **a**-type dislocations, not the other way around^20–22^. To overcome this hurdle, Wang et al*.*^18^, proposed the introduction of I_2_ BSFs through the decomposition of 60° misfit dislocation segments connected to TD arms. It was postulated that such dislocations are introduced by Matthews-Blakeslee glide of half-loops from the surface. The I_2_ BSF would eventually be annihilated through successive dislocation reactions, and the remaining TDs be connected to a hexagon of 60° misfit dislocation segments. However, this model lacks an explanation of how the **a**-type half-loops are introduced in the first place, given the lack of resolved shear stress.

An alternative explanation was suggested by us, involving instead the I_1_ intrinsic BSFs which are low-energy defects introduced during growth. We have shown that **a**-type TDs are introduced due to the overlap of I_1_ BSFs^16^. These BSFs can be delimited by either 1/6 < 20$\overline{2}$3 > Frank-Shockley PDs or prismatic stacking faults (PSFs). I_1_ BSF superposition leads to formation of closed domains in the form of distorted hexagonal prisms, eliminating the (0002) extra half planes (i.e. the Frank component of the PDs’ Burgers vector), leading to a significant reduction in localized elastic energy. Two cases of BSF domains were distinguished: In the first, the overlapping BSFs have mirror-related atom rings, i.e. opposite rigid-body translations (RBTs); in this case, no TDs are introduced. If, however, the BSFs have parallel RBTs, then TDs invariably nucleate pinned to these domains. However, in this early work, the actual mechanism of TD heterogeneous nucleation was not detailed.

Motivated by the high importance of tackling the excessive introduction of TDs, we need to fully elucidate the phenomenon of TD introduction from BSFs, i.e. whether I_1_ or I_2_ BSFs are involved, and if it is related to Matthews-Blakeslee glide for misfit relaxation^13^, or if it is growth-related. In this work, we describe the topological character of BSF domains, the defects that delimit them, and the actual mechanism of TD emanation from them. We do this for a range of samples comprising various III-nitride alloy heterostructures. We analyze the dislocation reactions leading to TD nucleation, and consider the influences of misfit strain and alloy fluctuations. Initially, transmission electron microscopy (TEM) observations are given, based on which, a topological analysis then yields the admissible defect configurations. The results are correlated to the compositional variations and elastic strain.

## Results

### Electron microscopy observations

**Figure 1:**
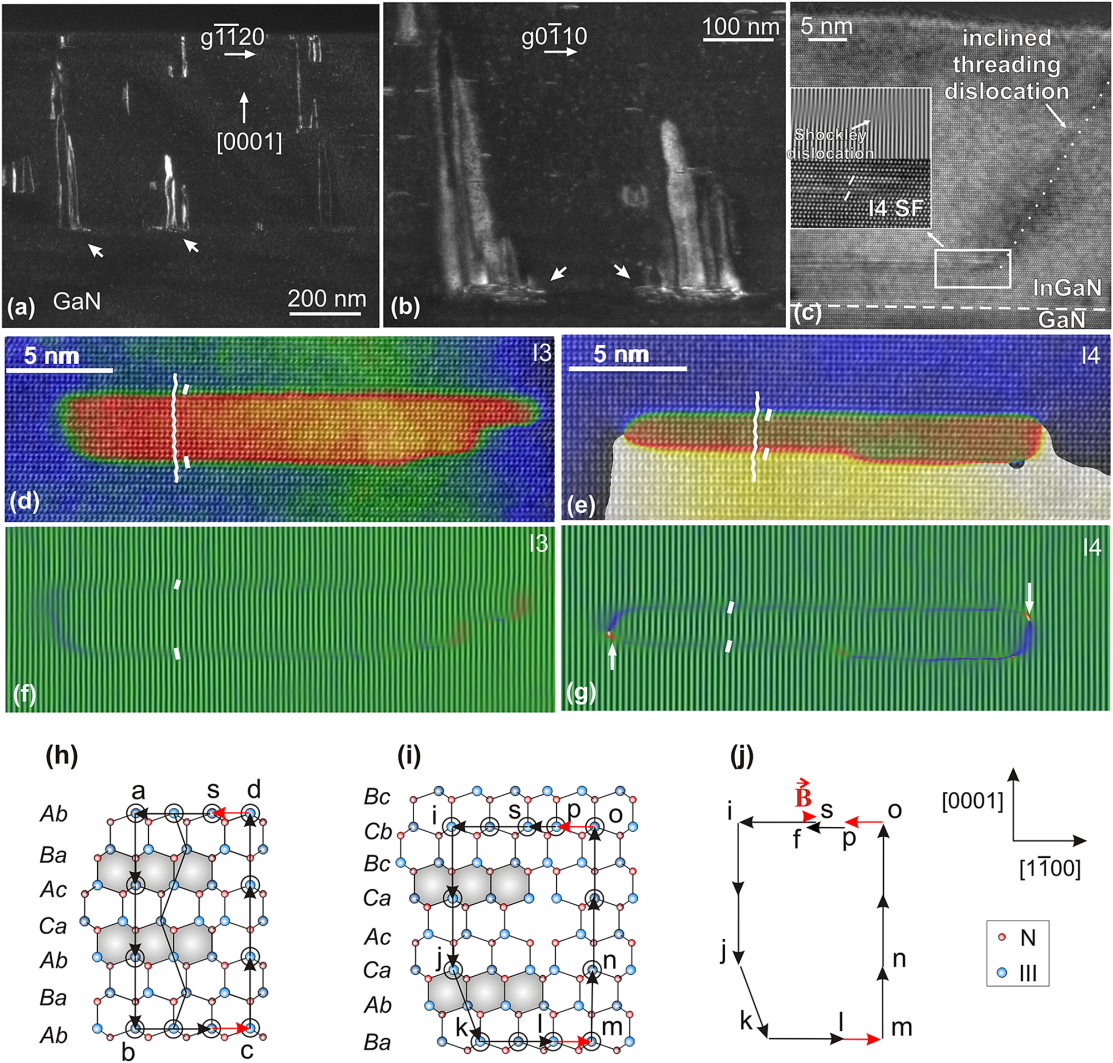
TEM and HRTEM images of emanating TDs and their nucleation sites. (**a**) Cross-sectional weak-beam TEM image obtained with *g*$\overline{1}\overline{1}$20 under *g*/3*g* conditions, showing **a**-type TD inverse half loops emanating at various heights along the growth direction in the In_0.02_Al_0.08_Ga_0.9_N:Mg film (misfit 0.017%). Arrows highlight the nucleation sites of two of them. (**b**) Magnification of the region highlighted by arrows in (**a**), now imaged with *g*0$\overline{1}$10. It can be discerned that the half loops nucleate at regions comprising overlapped defects. (**c**) Cross-sectional HRTEM image along [$\overline{1}\overline{1}$20], showing emanation of an inclined TD from the edge of a BSF domain in the In_0.19_Ga_0.81_N epilayer. The inset shows that the domain comprises two overlapped I_1_ BSFs with parallel stacking sequences (I_4_ configuration). (**d**) and (**e**) Cross-sectional HRTEM images along [$\overline{1}\overline{1}$20] of an I_3_ and an I_4_ BSF domain respectively, superimposed with their colored phase maps. Phase maps were obtained by geometrical phase analysis (GPA) using *g*1$\overline{1}$00. Stacking sequences are indicated. Both domains comprise I_1_ BSFs. (**f**) and (**g**) The corresponding Bragg-filtered images showing (2$\overline{2}$00) lattice fringes. Extra half-planes are indicated by arrows in (**g**). (**h**) and (**i**) Atomistic models of an I_3_ and I_4_ defect respectively (unrelaxed). Chair atom rings of the two I_1_ BSFs are shaded. Closed Burgers circuits have been indicated around their edges. ([$\overline{1}\overline{1}$20] projection; shading of atoms indicates distinct levels along the projection direction.) (**j**) Mapping of the closed circuit of (**i**) showing the closure failure **B** = **fs**. Black color denotes vectors on the ($\overline{1}\overline{1}$20) plane. Red vectors comprise an additional out-of-plane component equal to 1/6[$\overline{1}\overline{1}$20].

Our experimental observations are based on studies of several III-nitride alloy epilayers deposited on (0001) GaN either by molecular beam epitaxy (MBE) or metalorganic vapor phase epitaxy. In our samples, TDs that emanate from BSFs often form inverse half loops indicative of a heterogeneous nucleation phenomenon. This is illustrated in Fig. 1a showing several such defects in the MBE-grown In_0.02_Al_0.08_Ga_0.9_N:Mg epilayer. The particular half loops are of rather large size, with some exceeding 100 nm in height, although some small ones are also discernible. Careful examination of their nucleation sites points to BSF overlaps, in the manner described by Smalc-Koziorowska et al*.*^16^ (Fig. 1b). Figure 1c shows a high resolution TEM (HRTEM) image of an inclined TD, emanating from a BSF domain in the more strained In_0.19_Ga_0.81_ N epilayer, similar to what has been observed by Wang et al*.*^18^. It can be seen that the TD emanates from a region comprising not a single I_2_ BSF, but an overlap of two I_1_ BSFs with parallel stackings, as shown in the inset of Fig. 1c. Figures 1d–g illustrate cross-sectional HRTEM images of the two possible cases of I_1_ BSF overlap. In Fig. 1d, the BSFs have opposite RBTs, and so a closed prismatic domain is formed that is displaced relative to the matrix but with no dislocations required at its edges, as can be seen by the lack of extra-half planes in the Bragg-filtered image of Fig. 1f. The *g*1$\overline{1}$00 phase map shows that there is no phase change in the matrix, since the RBTs of the BSFs cancel out. This domain is crystallographically equivalent to the I_3_ BSF^23^. Figure 1e illustrates the case whereby the overlapping I_1_ BSFs have parallel RBTs, which we have termed an I_4_ defect^16^. It is seen that now there is a net phase change in the surrounding matrix, and extra half-planes are observed on either side of the domain (Fig. 1g), showing it has dislocation content. We also note the lack of (0002) extra half-planes in both domains. Figures 1h,i illustrate unrelaxed atomistic models of the I_3_ and I_4_ defects. In the I_3_ case, there is always an odd number of layers of boat-shaped atomic rings between the mirror-related chair-shaped rings, with the minimum being just one layer^21^. In I_4_, there is an even number of layers with the minimum equal to two.

**Figure 2:**
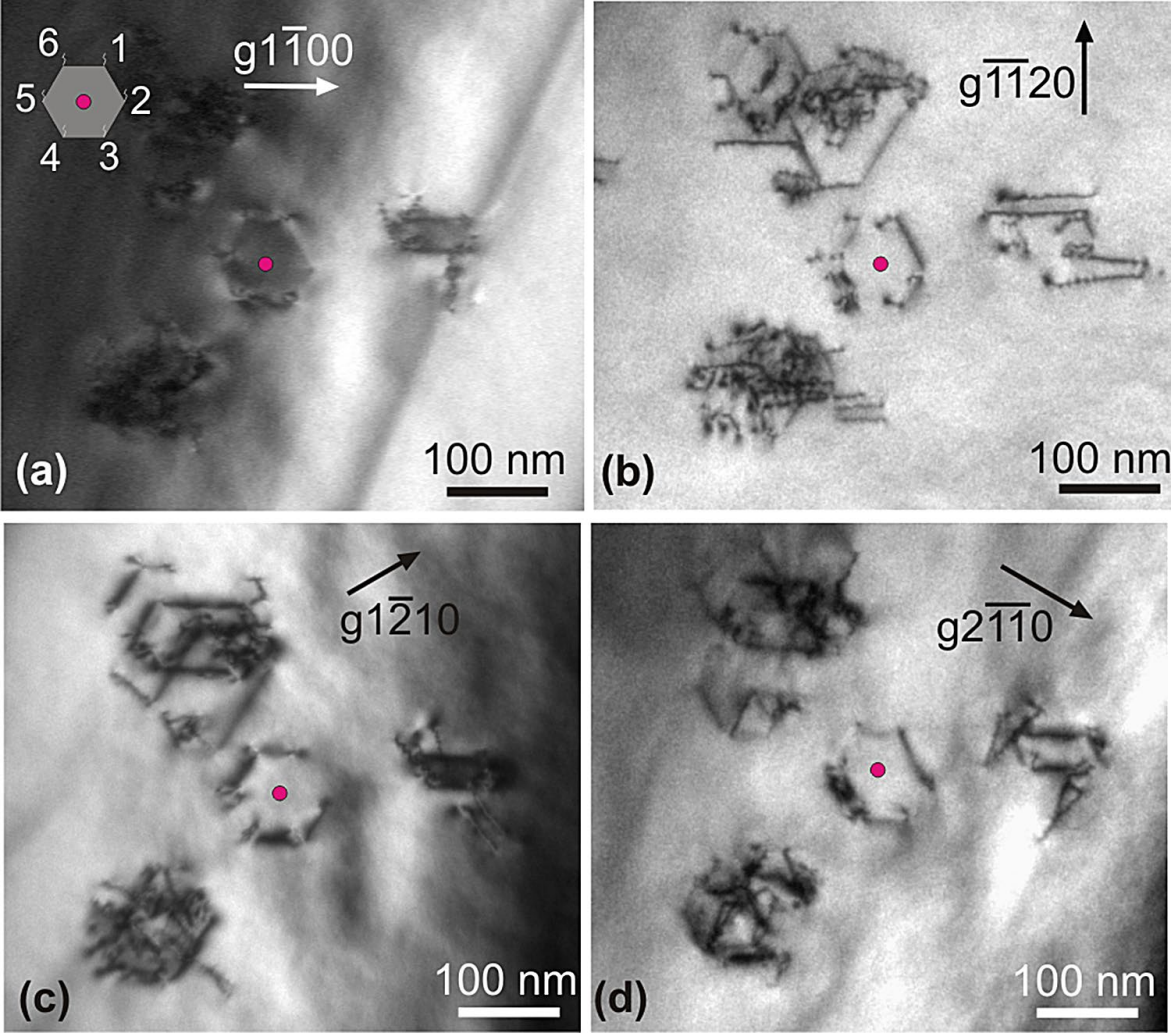
Plan-view [0001] two-beam bright field TEM images of a hexagonal stacking fault domain recorded with *g*(1$\overline{1}00$) and the three *g*{$\overline{1}\overline{1}$20} reflections. In (**a**), dark BSF contrast is observed. TDs are seen to emanate from the nodes of the hexagon (more clearly visible in (**b**)). Hexagon sides are aligned along < 1$\overline{1}00$ > directions. Opposite sides of the hexagon comprise screw PDs with antiparallel Burgers vectors as manifested by their simultaneous alternating invisibility in (**b**–**d**).

So far, we have shown that (i) domains comprise I_1_, not I_2_ BSFs, (ii) only I_4_ domains can have defect content, and (iii) the emanating TDs can form inverse half-loops. We now concentrate on the mechanism of TD nucleation, performing a detailed analysis of such domains from plan-view TEM observations. Figure 2 illustrates domains with emanating TDs, observed along [0001] in the In_0.02_Al_0.14_Ga_0.84_N:Mg epilayer. They have diameters below 100 nm in this sample. In Fig. 2a, taken with *g*{1$\overline{1}$00}, they exhibit characteristic dark contrast due to the 1/3 < 10$\overline{1}$0 > in-plane component of the RBT. We also note that the sides of the domains, and hence their PDs, are aligned along < 1$\overline{1}$00 > (*m*-type) directions. Figures 2b–d illustrate images recorded with *g*{$\overline{1}\overline{1}$20} reflections, and the BSF contrast now disappears. Indeed, intrinsic BSFs are invisible when any of the *g*{$\overline{1}\overline{1}$20} reflections is employed in two-beam diffraction contrast, according to the **g.p** invisibility criterion, where **p**_*i*_ = 1/6 < 20$\overline{2}$3 > is the RBT, **g** is the reciprocal lattice vector, and **g.p** = 0 or integer for invisibility.

For the topological characterization of the dislocations, the **g.b** = 0 invisibility criterion is commonly applied, where **b** is the Burgers vector. An **a**-type 1/3 < $\overline{1}\overline{1}$20 > TD could be rendered almost invisible with one *g*{1$\overline{1}$00} reflection. However, even for **g.b** = 0, large values of the product **g.b × u** are obtained (where **u** is the line direction of the dislocation), signifying strong residual contrast (see Supplementary Table S1 online). The overlap with the BSF’s dark contrast further impedes the topological analysis of these TDs. Hence, we concentrate only on the PDs, using *g*{$\overline{1}\overline{1}$20} reflections since (i) the BSF contrast fully disappears and is not a complicating factor, and (ii) for their **u** =  < 1$\overline{1}$00 > line directions, residual contrast is not a problem, i.e. they can be rendered fully invisible. In Fig. 2b, the PD segments between TDs 3–4 and 6–1 are both out of contrast with *g*$\overline{1}\overline{1}$20, but are visible with the other two *g*{$\overline{1}\overline{1}$20} vectors (Fig. 2c,d). This condition is repeated for the other two pairs of hexagon sides. In Fig. 2c, taken with *g*1$\overline{2}$10, the PDs between TDs 1–2 and 4–5, are invisible with no residual contrast, and in Fig. 2d, the remaining PDs 2–3 and 5–6 are out of contrast. Hence the in-plane Burgers vectors are ± 1/3[1$\overline{1}$00] for PDs 3–4 and 6–1, ± 1/3[10$\overline{1}$0] for PDs 1–2 and 4–5, and ± 1/3[01$\overline{1}$0] for PDs 2–3 and 5–6. What is particularly interesting is that, based on their contrast, all PD segments are of screw type. This raises an important question regarding the mechanism of their introduction, since screw dislocations cannot relieve in-plane misfit strain. In previous work^16,18^, it was postulated that the energetically unfavorable introduction of lattice TDs from PDs could be facilitated by the relief of elastic strain energy but this does not seem to be the case. Hence TD emanation from nodes with PDs should be a result of dislocation reactions and nodal balance.

**Figure 3:**
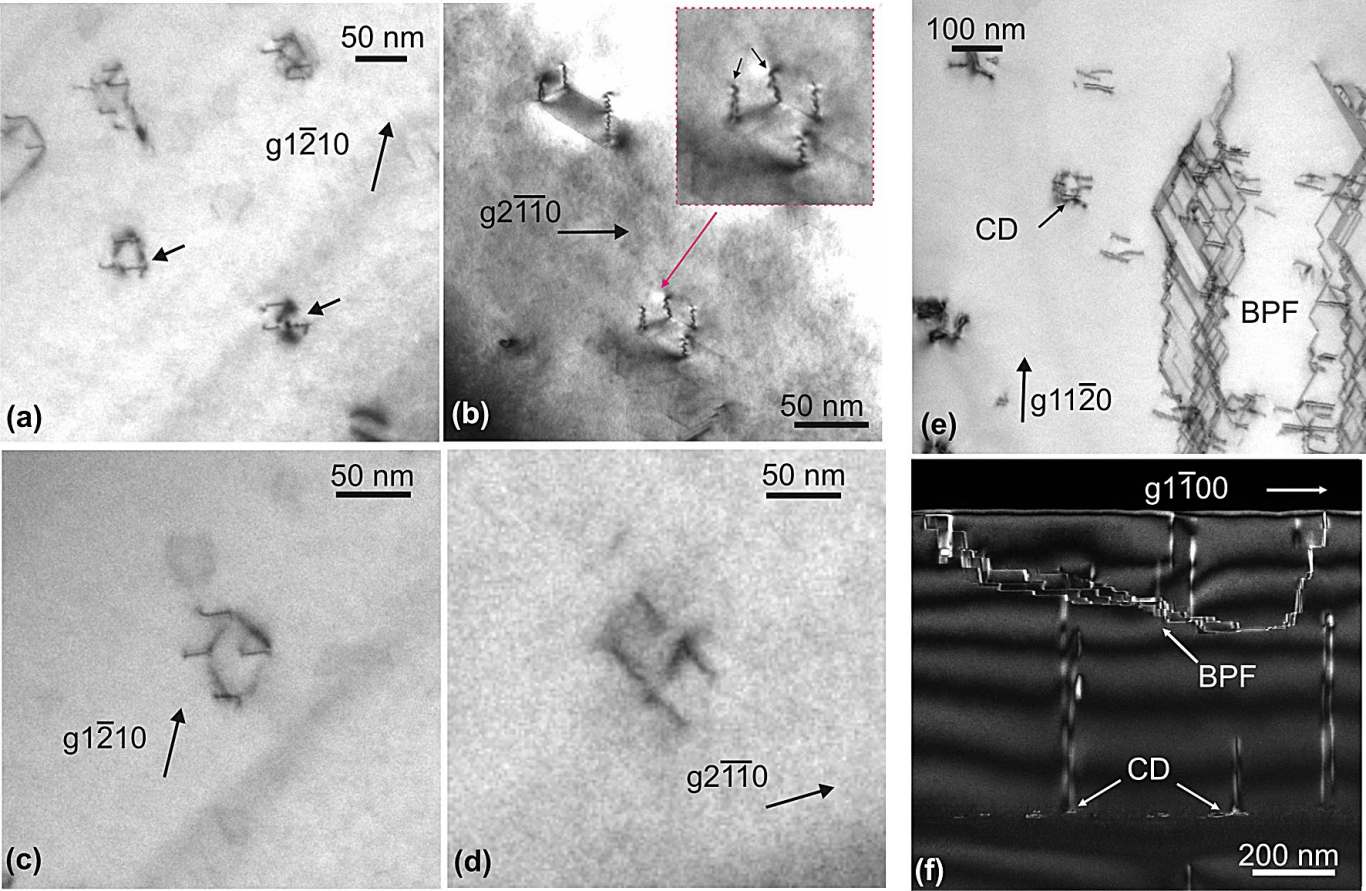
BSF domain morphologies and BSF/PSF folds. (**a**,**b**) Plan-view bright-field TEM images showing stacking fault domains with triangular and trapezoidal morphologies respectively. The inset of image (**b**) shows the trapezoidal domain in magnification, with arrows indicating **a**-type TDs with opposite Burgers vectors. (**c**) and (**d**) Plan-view images of the same rhombic domain. All PDs are visible with *g*1$\overline{2}$10 in (**c**), whereas, in (**d**), two PDs, along [01$\overline{1}$0] are in extinction with *g*2$\overline{1}\overline{1}$0. (**e**) and (**f**) Bright–field plan-view and dark-field cross-sectional two-beam images respectively of the large open domain with BSF/PSF folds (BPF). Some smaller closed domains (CD) are also indicated.

In Fig. 3, other triangular (Fig. 3a), trapezoidal (Fig. 3b), and rhombic (Fig. 3c,d) BSF domains are illustrated again showing extinctions of their sides with diffracting vectors normal to them, always consistent with screw PD character. In Fig. 3c,d, the same rhombic domain is depicted. In Fig. 3c, all PDs are visible since the employed diffracting vector is inclined whereas, in Fig. 3d, the two sides that are normal to the diffracting vector are out of contrast. Apart from the closed BSF domains, we also observed some much larger BSF/PSF folds, extending over few microns (Fig. 3e,f). In this configuration the stacking fault is folding back and forth between PSF and I_1_ BSF (Fig. 3f). They too are formed during growth and end at the epilayer surface^24^.

### Partial dislocations of stacking fault domains

Our experimental observations have clearly demonstrated that **a**-type TDs can emanate from BSF domains that are delimited by screw PDs along *m*-directions. Such BSFs can be introduced due to double positioning or due to deviations from stoichiometry during growth. For example, a low growth temperature could promote them by reducing adatom mobility, or their introduction can be related to nonstoichiometric flux ratios^25^.

We now follow a “bottom-up” description of the introduction of TDs from BSF prismatic domains, consistent with the overlapping formation of the I_1_ BSFs. Unrelaxed atomistic models of the I_3_ and I_4_ defects were illustrated in Fig. 1h,i respectively. In Fig. 1h, a closed Burgers circuit *sabcds* around the edge of the I_3_ fault shows no closure failure. Another such circuit *sijklmnops,* drawn around I_4_ in Fig. 1i, shows closure failure with Burgers vector **B** = **fs** = 1/6[1$\overline{1}$00] + 1/6[$\overline{1}\overline{1}$20] = 1/3[0$\overline{1}$10] when mapped to *sijklmnopf* in the reference space (Fig. 1j), i.e. a Shockley-like PD delimits the I_4_ defect. This PD has the same Burgers vector as the Shockley PD but is situated between the I_1_ BSFs in an analogy to zonal dislocations^26^.

**Figure 4:**
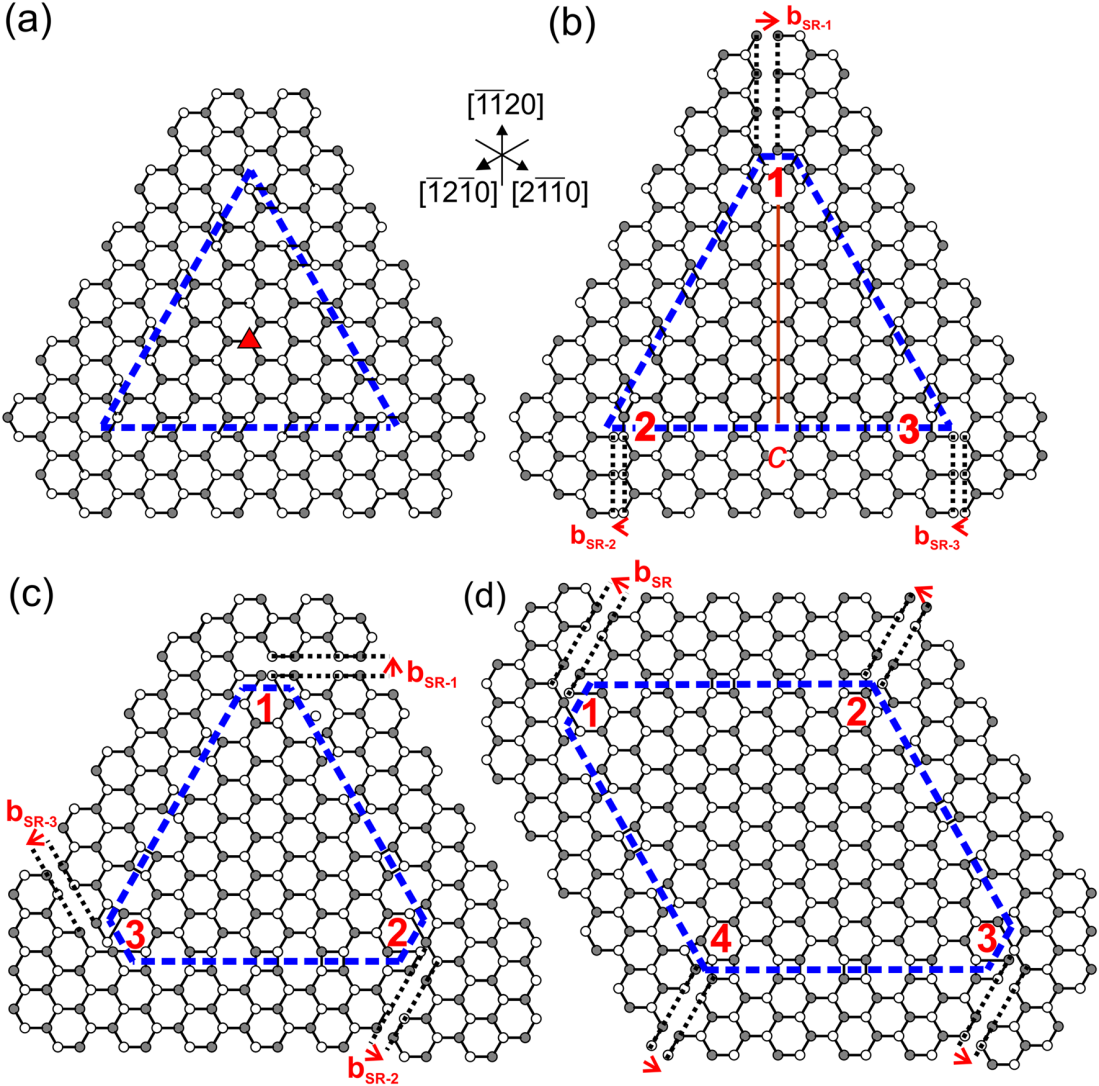
(**a**) A closed triangular configuration of Amelinckx PSFs with no dislocation. The domain is consistent with threefold symmetry, and the threefold axis is indicated by a red triangle. PSF planes are indicated by dashed blue lines. (**b**) Unrelaxed triangular domain whereby the Amelinckx PSFs of facets 1–2 and 1–3 are related by the *c*_(1–100)_ glide-mirror (indicated by a red line). Stair-rod dislocations at PSF facet junctions are shown using Volterra cuts indicated by dashed black lines. Their Burgers vectors **b**_SR*-j*_ are shown in red. The PSF of facet 2–3 has Drum character. The Burgers vectors are **b**_SR-2_ = **b**_SR-3_ = 1/6[1$\overline{1}$00] = -**b**_SR-1_/2. (**c**) A triangular arrangement of Drum PSFs forming 60° demi-stepped facet junctions with stair-rod PD character. The Burgers vectors are **b**_SR*-j*_ = 1/6 < $\overline{1}\overline{1}$20 > . (**d**) Rhombic arrangement of Drum PSFs.

Given the complete absence of Frank-Shockley PDs, we next examine the admissible {$\overline{1}\overline{1}$20} PSFs around the BSF domain and the associated stair-rod PDs. One PSF type has the same RBT **p**_*i*_ as the I_1_ BSF, and is known as Amelinckx PSF^27^. Then the facet junctions of the prismatic domain do not require defect character. For example, Fig. 4a illustrates a trigonal configuration of Amelinckx PSFs, whereas the 120° PSF facet junctions have been treated elsewhere^28^. It is also possible that the PSFs are of Drum type^29^, which is the low-energy PSF and has RBT **p**_D-*j*_ = ½ < 01$\overline{1}$1 > ^30^. Figure 4b shows an alternative to Fig. 4a, whereby one facet junction could exhibit stair-rod PD character with 1/3 < 1$\overline{1}$00 > Burgers vector, since the two Amelinckx PSFs are now related by the *c*_{1–100}_ glide-mirror. The facet vertical to this mirror plane then easily becomes a Drum PSF, with concurrent introduction of 1/6 < $\overline{1}$100 > stair-rod PDs at the facet junctions with the Amelinckx PSFs (junctions 2 and 3 of Fig. 4b).

Τhe I_1_/Drum facet junctions have Burgers vectors **b**_SR-*ij*_ = **p**_D-*j*_ – **p**_*i*_ = 1/6 < 01$\overline{1}$0 > . Since, these junction lines are along < 10$\overline{1}$0 > directions, they are screw type stair-rod PDs. At the Drum PSF facet junctions we have edge-type stair-rod PDs with **b**_SR*-j*_ = 1/6 < 2$\overline{1}\overline{1}$0 > Burgers vectors (i.e. exactly half a lattice vector), as has been detailed elsewhere^28^. This is shown in Fig. 4c with an atomic supercell for the triangular domain. For the triangular and hexagonal domains, the orientation of **b**_SR*-j*_ changes at each facet junction. For the rhombus (Fig. 4d), it stays the same but can be antiparallel. As we will show later, the presence of stair-rod PDs at the junctions between BSFs and PSFs in I_4_ domains facilitates the nucleation of **a**-type TDs. Moreover, a BSF domain can contain either stair-rod or Shockley-like PDs but not both of them together.

### Topological analysis of TD nucleation at BSF domains

A single I_1_ BSF bounded by a Frank-Shockley PD loop can overlap with a second such BSF that exhibits the opposite RBT, as illustrated in Supplementary Note S1 and Fig. S1 online, so that the net dislocation content is zero and the I_3_ defect is constructed. If the side facets are Drum PSFs instead of Amelinckx ones, the facet junctions, will have stair-rod character but the net Burgers vector will remain zero.

**Figure 5:**
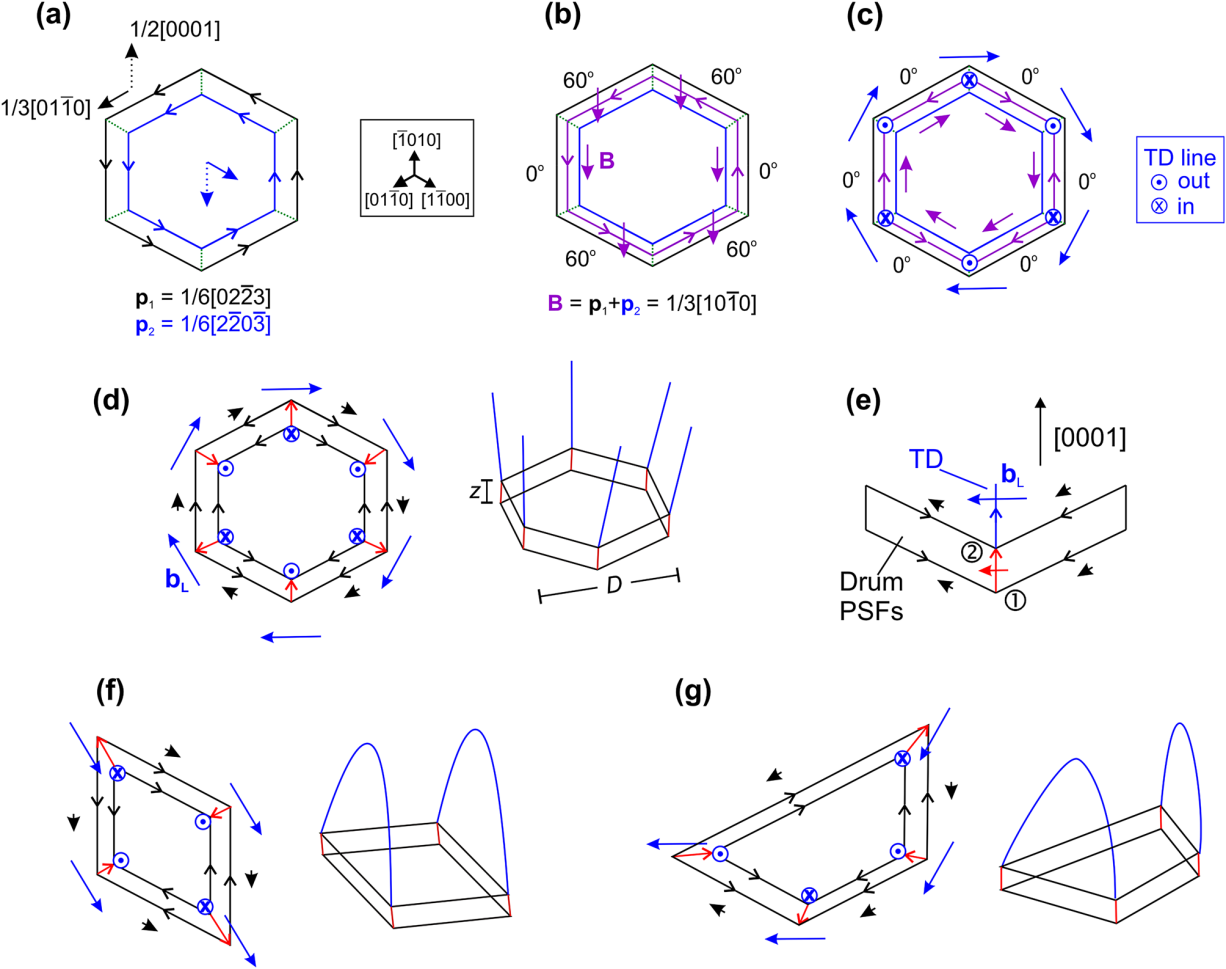
Schematics of line defect topological properties and nodal balances. (**a**) Perspective view along [0001] of two overlapped I_1_ BSFs (black:bottom and blue:top) with RBTs **p**_1_ = 1/6[02$\overline{2}$3] and **p**_2_ = 1/6[2$\overline{2}$0$\overline{3}$] (same sense of BSF atom rings). The out-of-plane Frank components are denoted by dashed vectors. The loops’ sides are along *m*-directions, and the senses of the Frank–Shockley PDs are indicated. (**b**) Net result of (**a**) giving an I_4_ domain delimited by a Shockley-like PD loop. The Burgers vector **B** and the line direction of the loop are indicated in purple color. (**c**) Replacement of the 60° loop segments by 0° screw ones and concurrent emanation of six **a**-type lattice TDs. Line directions and Burgers vectors are shown in purple for PDs and in blue for TDs. (**d**) Hexagonal I_4_ domain comprising Drum PSFs, showing emanation of six **a**-type TDs. Vectors are blue for TDs, red for 1/6 < $\overline{1}$2$\overline{1}$0 > stair-rods, and black for 1/6 < $\overline{1}$100 > stair-rods. (**e**) Side view of one facet junction, illustrating the emanation of a TD with **b**_L_** = **1/3[$\overline{1}$2$\overline{1}$0] (blue vector) from node 2 as a result of the reaction between stair-rod PDs. Vectors are drawn to scale. Red: 1/6[$\overline{1}$2$\overline{1}$0] stair-rod. Black: 1/6[$\overline{1}$100] and 1/6[01$\overline{1}$0] stair-rods. (**f**) Rhombic I_4_ domain with two TD half-loops of the same Burgers vector. (**g**) Trapezoidal I_4_ domain with two TD half-loops of distinct Burgers vectors.

In the I_4_ case, if say the first I_1_ BSF is described by **p**_1_ = 1/6[02$\overline{2}$3], the second one may be described by **p**_2_ = 1/6[2$\overline{2}$0$\overline{3}$] as shown in Fig. 5a for a hexagonal prism with facet junctions along the *m*-directions. Then the BSF domain introduces a net RBT of the matrix crystal equal to 1/3[10$\overline{1}$0], accommodated by a **B** = 1/3[10$\overline{1}$0] Shockley-like PD, as shown in Fig. 5b. This PD has two screw and four 60° mixed segments. However, the coexistence of mixed and screw PD segments in a single loop would require different deformations of the PSF structural units. On the contrary, our plan-view TEM observations have shown that all PDs segments are screw type and each segment has a different Burgers vector. So, it appears that a single PD loop is not the most stable physical model. Figure 5c shows that the model of six PD segments with screw character can only be attained after introduction of six lattice TDs emanating from the [0001] facet junctions. In particular, the 60° PD segments are replaced according to equations such as 1/3[10$\overline{1}0]$ (60°) = 1/3[0$\overline{1}$10] (0°) + 1/3[11$\overline{2}$0] (90°). So, edge-type TDs emanate from the dislocation nodes into the crystal above the BSF domain. The whole configuration is nicely consistent with hexagonal symmetry.

We may then consider why this configuration of Fig. 5c would be preferred over Fig. 5b. On the basal plane, the overall energy is obviously less since we have screw segments only, instead of the higher energy mixed ones. On the other hand, such a model would normally not be considered energetically favorable, since the lattice TDs bear a high self-energy. However, we should take into account that this is a sequential (bottom-up) growth phenomenon and not a post-deposition dislocation reaction. Therefore, the TDs are first nucleated as geometrically necessary short segments that are then replicated during growth just like the TDs coming from the substrate. On this basis, the energetical comparison of the two configurations is treated in the Discussion.

Moreover, extra energy promoting further the TD nucleation can be provided by introducing Drum PSFs instead of Amelinckx ones. The accommodation of six such PSF facets in a hexagonal prism is illustrated in Fig. 5d, and nodal balance is detailed in Fig. 5e. Along each PSF facet, the Shockley-like 1/3 < 01$\overline{1}$0 > screw PD is dissociated into two screw 1/6 < 01$\overline{1}$0 > stair-rod PDs (which is also energetically favorable). In Fig. 5e, the nodal balance at node no. 1 is 1/6[$\overline{1}$100] + 1/6[01$\overline{1}$0] = 1/6[$\overline{1}$2$\overline{1}$0]. At node no. 2, the balance between in-coming and out-going dislocations is 1/6[$\overline{1}$100] + 1/6[01$\overline{1}$0] + 1/6[$\overline{1}$2$\overline{1}$0] = **b**_L_ = 1/3[$\overline{1}$2$\overline{1}$0], giving the Burgers vector of the geometrically necessary TD. The energetics of this model are also discussed later.

It is noted that, in Fig. 5c,d, TDs emanating from opposite vertices have both opposite line directions and Burgers vectors, and hence are in fact parallel. Since antiparallel TD pairs do not appear, formation of inverse TD half loops from hexagonal prisms is excluded (and similarly for the trigonal prism). On the other hand, the rhombic and trapezoidal morphologies can yield inverse TD half-loops, as shown in Fig. 5f,g. The rhombus yields two TD half-loops of the same Burgers vector (cf. also Fig. 4d, while the trapezium gives two half-loops of distinct Burgers vectors. An experimental example of a trapezoidal BSF domain has been illustrated in Fig. 3b. TD half loops are not formed immediately upon TD introduction, especially in the case of large BSF domains. In Fig. 1a we observe many half-loops starting at the BSF domains with various lengths. It indicates that half-loop formation occurs later during growth, when by either mutual attraction or by the strain present in the epitaxial layer, TDs bend towards each other.

### EELS analysis of indium distribution at stacking fault domains

In order to check if the domains are related to compositional changes in the alloy epilayers, electron energy loss spectroscopy (EELS) was performed. Low loss EELS was conducted in the In_0.19_Ga_0.81_N epilayer for the detection of chemical inhomogeneities related to the BSF domains. In the case of In_*x*_Ga_1-*x*_N ternaries, the energy position of the plasmon peak is related to the In-content through a parabolic version of Vegard’s law. In particular, a ~ 0.4 eV shift of the plasmon peak is expected for every 10% change in In-content^31^. For the plasmon peak analysis, dual-EELS was performed, acquiring simultaneously the zero-loss and the plasmon peak regions. All plasmon peak spectra were aligned to the zero-loss peak in order to eliminate possible energy drifts during acquisition, so as to improve the precision in the evaluation of the plasmon peak position. Fourier logarithm deconvolution was applied to the aligned spectra in order to remove possible plural scattering, and the position of the plasmon peak was determined by fitting a Gaussian to the experimental data. The sample thickness (*t*) was evaluated by low-loss EELS according to $\frac{t}{\lambda } = {\ln}\left( {\frac{{I_{t} }}{{I_{0} }}} \right)$, where λ is the inelastic mean free path, *I*_t_ is the intensity of the entire spectrum and *I*_0_ the intensity of the zero-loss peak. It was estimated to be ~ 10 nm by this method.

**Figure 6:**
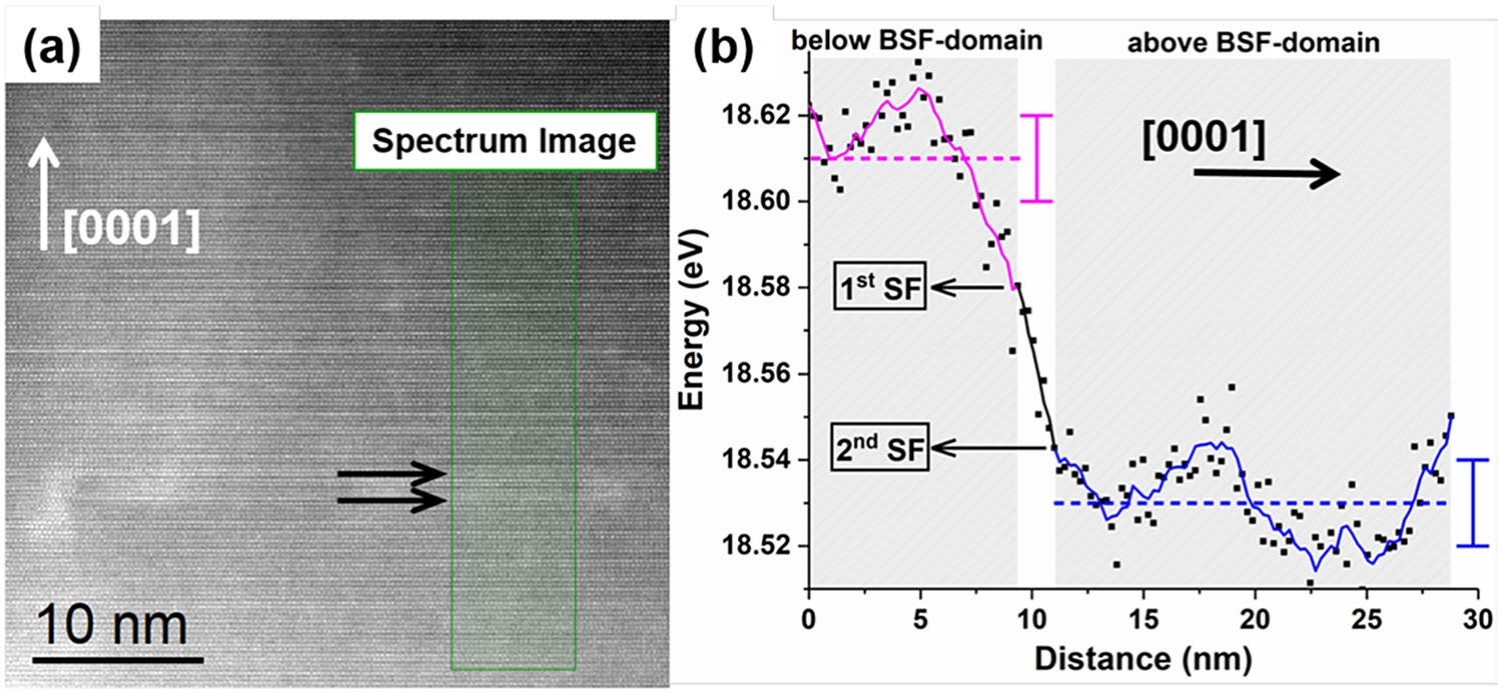
(**a**) ADF HRSTEM image showing a BSF domain with emanating TD. The green box annotates the area where EELS was conducted (black arrows annotate the position of the two BSFs). (**b**) The corresponding profile of the center of the fitted plasmon peak shows a clear energy shift after crossing the BSF domain, revealing 2.5% increase in the In-content above the defect. The dashed lines show the mean values of the raw data of each area.

Figure 6 shows a representative annular dark field (ADF) high resolution scanning TEM (HRSTEM) image of a BSF domain, whereby EELS analysis was conducted crossing the domain along the [0001] direction (green box). The extracted profile of the center of the fitted plasmon peak shows a clear energy red shift (~ 0.1 ± 0.01 eV) at the area above the defect (blue) with respect to the area below (purple). Since plasmon energy shifts can also be related to elastic strain variations, GPA was also conducted but showed no measurable strain. Hence the observed energy shift is attributed to a ~ 2.5% increase in indium concentration above the domain in this case. Such small variations of the indium content when crossing the domains were generally observed but were not systematic. Since the In_0.19_Ga_0.81_N layer was only 12% relaxed through introduction of (**a** + **c**) misfit dislocations^15^, the observed changes in the indium content may be attributed to local alloy compositional fluctuations on account of the compositional pulling phenomenon, and not to a strain relaxation process^32–34^. Also, we did not find by EELS that the BSFs themselves or the emanating TDs were enriched in indium.

## Discussion

Identification of mechanisms of TD nucleation is crucial for III-nitride epilayers. In this case, the correlation of TD introduction to I_1_ BSFs is a general mechanism that is observed in various alloy epilayers. Previous observations have pointed out this correlation, but the mechanism of TD emanation was not elaborated in detail. Moreover, it was postulated that strain could be promoting the phenomenon. Our work has pointed out important features that largely elucidate this mechanism. We have excluded the I_2_ BSF, which is predominantly a strain-induced fault, as a source of these TDs. On the contrary we observe them to be introduced due to the overlap of I_1_ BSFs. Such BSFs are growth faults of much lower energy^23^, and far more abundant than I_2_ ones. However, their delimiting Frank-Shockley PDs have very large Burgers vector (almost equal in magnitude to the lattice constant *a*), and the largest part is along the growth direction, i.e. the *c*/2 Frank component. It appears that instead of formation of a Frank-Shockley PD loop, the BSF is delimited by PSFs and this eliminates the (0002) extra half plane^24^. However, this requires a second I_1_ BSF, in order to close the domain. In order to ascertain the contribution of formation energy to the phenomenon, five BSFs, i.e. I_1_, I_2_, I_3_, I_4_ and extrinsic (E), were atomistically modeled under periodic boundary conditions. For the I_3_ and I_4_, calculations were performed for the minimum spacing between the layers of chair-shaped atom rings, as depicted in Fig. 1h,i. The sizes of the employed models ranged from 56 atoms, for the I_1_ BSF, to 88 atoms, for the E BSF. Results are listed in Table 1 from where it is seen that the I_3_ and I_4_ defects have practically the same formation energy, slightly smaller than that of I_2_.

**Table 1:** Calculated BSF formation energies for GaN in erg/cm^2^.

I_1_	I_2_	I_3_	I_4_	Extrinsic
17.8	37.8	35.5	35.4	58.1

Our experimental observations are inconsistent with the model of Wang et al.^18^ in several aspects. The observation of inverse TD half loops dismisses Matthews-Blakeslee dislocation glide, since the half loops are oriented in the opposite way. This demonstrates that, in our samples, TD introduction is a bottom-up process rather than a top-down one. We have also established that the PDs of the domains, are aligned along *m*-type < 1$\overline{1}$00 > line directions, not < $\overline{1}\overline{1}$20 > , and they are of screw type, not mixed or edge. The observed contrast of these dislocations under two-beam TEM imaging using *g*{$\overline{1}\overline{1}$20} reflections, shows it is crystallographically impossible for them to be **a**-type dislocation segments on the basal plane, but only PDs. Moreover, the emanation of TDs from triangular domains cannot be explained by their mechanism, which requires always an even number of emanating TDs. Besides, in our samples, domains with emanating TDs have BSF character, whereas they claim that the BSF character is annihilated in the final configuration. We have to exclude strain as the reason for the formation of these BSF domains and associated TDs. We observe them in epilayers that are almost fully relaxed as well as in ones that retain some residual elastic strain. EELS analysis showed small non-systematic stoichiometry increase when crossing such domains in InGaN epilayers. However, since the domains are bounded by screw type PDs, this excludes the possibility of the domains themselves contributing to strain relaxation. On the other hand, TD bending could be attributed to elastic strain relaxation^35,36^, and Fig. 1c illustrated such a case. If not due to the residual elastic strain, introduction of BSF domains should be attributed to the growth conditions, such as for example conditions promoting a reduced adatom mobility, incomplete adlayer surface coverage, or enhanced adatom desorption^25,37^. Under such conditions, I_1_ BSF superposition could be promoted by the need to eliminate the (0002) extra half planes along the growth direction. Indeed, there are rather few experimental reports of Frank-Shockley PDs, and these are predominantly close to the heteroepitaxial interface^38^.

**Figure 7:**
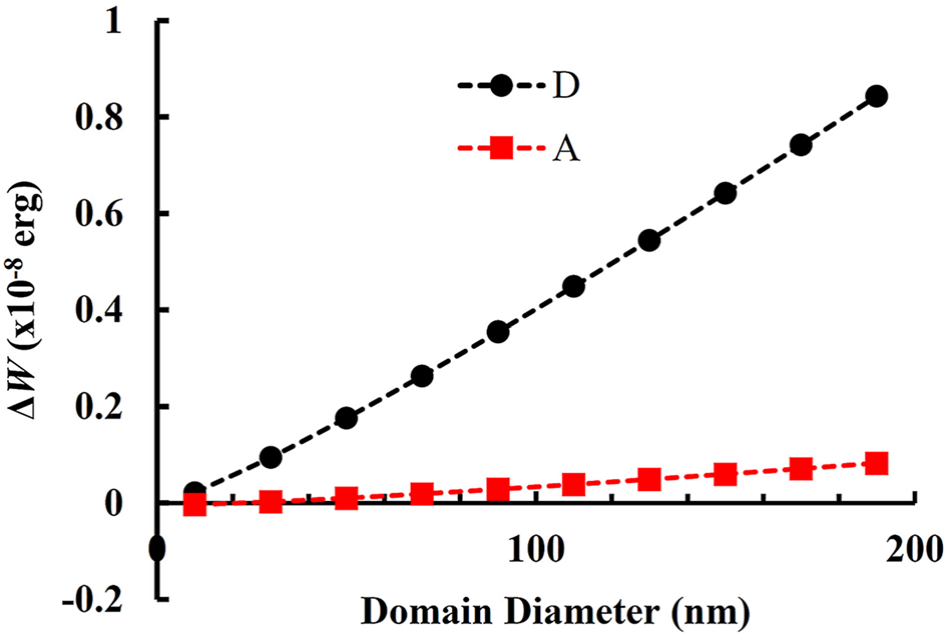
Graphs of *ΔW*_A_ and *ΔW*_D_ relative to I_4_ domain diameter, corresponding to the energetical comparison between the configuration of Fig. 5b–d respectively. The graphs are drawn for domain height *z* = 1 nm.

The proposed mechanism considers the superposition of I_1_ BSFs with parallel RBTs and introduces the TDs as geometrically necessary defects. Further insight into its stability can be provided by comparing the configuration of a single Shockley-like dislocation around the domain (Fig. 5b) to those of Fig. 5c,d with emanating TDs and screw PD segments. A qualitative understanding can be obtained by a simple energetic approach using isotropic elasticity, and we performed such calculations for a closed hexagonal domain. The calculation details are given in the Supplementary Note S2 online. The energy difference, *ΔW*_A_, between the configurations of Fig. 5b,c is

1 $$ \Delta W_{{\text{A}}} = W_{{{\text{loop}}}} {-}\left( {W_{{\text{s}}} + W_{{{\text{TD}}}} } \right) $$

where W_*loop*_ is the total energy of the Shockley-like PD loop of Fig. 5b, *W*_s_ is the total energy for the screw PD segments of Fig. 5c, and *W*_TD_ the self energy of the six TDs. A positive energy difference *ΔW*_A_ means that the configuration of Fig. 5c has lower energy than that of Fig. 5b and is more preferable. In a first approximation we may regard the TDs as small segments of very small height since, after nucleation, their replication during growth does not pertain to what has already taken place on the basal plane, i.e. at the level of the domain. The minimum height of an I_4_ domain, is related to the minimal distance between two BSFs which is approximately 1 nm, so a reasonable assumption for the TD segments is equal to that. Figure 7 illustrates the diagram of Δ*W*_A_ with respect to domain diameter *D*. It is seen that, under these assumptions, *ΔW*_A_ is slightly positive, making such a domain energetically favorable compared to a single Shockley-like PD loop across the whole diameter range.

We next consider the configuration of Fig. 5d instead of Fig. 5c, and compare it to that of Fig. 5b. The energy difference between the configurations of Fig. 5b,d is

2 $$ \Delta W_{{\text{D}}}  = W_{{{\text{loop}}}} {-}\left( {W_{{{\text{sr}}}}  - W_{{{\text{int}}}}  + W_{{{\text{TD}}}} } \right) + \left( {zD/{\text{2}}} \right)\left( {E_{{\text{A}}}  - E_{{\text{D}}} } \right) $$

where *W*_sr_ is the total energy of the stair-rod dislocations, *W*_int_ is the energy of the repulsive interaction between stair-rod pairs, and *z* is the domain height as indicated in Fig. 5d. Equation (2) also comprises the energy difference between the Amelinckx and Drum PSFs taken equal to $E_{{\text{A}}}$-$E_{{\text{D}}}$ = 30 meV/A^2^ for binary GaN material^30^. As seen in Fig. 7, shown for domain height *z* = 1 nm, the configuration of Fig. 5d is now much more favorable compared to Fig. 5b across the whole diameter range, i.e. Drum PSFs promote the TD nucleation considerably. Further increasing the domain height makes the configuration of Fig. 5d even more favorable. More detailed insight into the mechanism should be provided by energetical simulations.

### Conclusions

Emanation of TDs from BSF domains has been attributed to the superposition of I_1_ BSFs with parallel rigid body translations. Plan-view TEM observations have shown that such closed prismatic domains are delimited by screw PDs along < 1$\overline{1}$00 > lines, and hence they do not relieve residual elastic strain. Dislocation glide from the surface also does not appear to be directly related to the phenomenon since inverse orientation of the TD half-loops was observed. When the growth conditions favor the introduction of intrinsic I_1_ BSFs, they are more likely to be surrounded by PSFs rather than being terminated by Frank-Shockley PDs, since in this way the high energy Burgers vector component **c**/2 is avoided. This requires introduction of a second I_1_ BSF terminating the PSFs for eventual closure of the domain. Formation of Shockley-like dislocations surrounding such a closed domain depends on the number of monolayers between the two BSFs. Our proposed model imposes the introduction of the TDs as geometrically necessary defects for the coexistence of crystallographically equivalent morphological variants of the PSFs around the domain, with all the PDs being of screw character. The mechanism appears to be energetically favorable, particularly for the case of Drum-type PSFs, and to explain well all the experimentally observed configurations of BSF domains.

### Methods

#### Sample growth

We present here results from MBE-grown In_0.02_Al_0.08_Ga_0.9_N:Mg, In_0.02_Al_0.14_Ga_0.84_N:Mg and 12% relaxed In_0.19_Ga_0.81_N epilayers. The misfit to GaN was 0.017%, 0.12% and 1.8%, respectively. The InAlGaN layers were parts of a blue laser diode heterostructure, being used as electron blocking layers or p-type claddings. All epilayers were grown at 650 °C under indium-rich conditions. The InAlGaN layers were deposited in a customized VG V90 MBE reactor equipped with two Veeco radio frequency plasma sources. The InGaN layer was deposited in a Gen20 Veeco reactor equipped with a high growth rate plasma source. Growth details are given elsewhere^39–41^.

### Transmission electron microscopy

TEM, scanning-TEM (STEM), and HRTEM observations were performed using a 200 kV TECNAI G2 F20 S-TWIN and a 300 kV aberration-corrected FEI Titan cubed 80–300 microscope from FEI (ThermoFisher). HRSTEM and EELS were conducted on a probe-corrected and monochromated FEI Titan G2 60–300 kV. HRSTEM observations were performed with a convergence angle of 24 mrad, using simultaneously ADF, high angle ADF (HAADF), and annular bright field (ABF) detectors. Spatial resolution was approximately 0.08 nm. EELS was performed using a Gatan Quantum 965 imaging filter with energy dispersion 0.1 eV/channel and energy resolution 1.2 eV. Observations were performed in cross section along [$\overline{1}\overline{1}$20] and [1$\overline{1}$00], and in plan-view along [0001]. Specimen preparation was performed by mechanical grinding, followed by ion milling in the Gatan PIPS. GPA and circuit mapping were used for defect characterization^42–44^.

### First-principles calculations

Atomistic calculations of BSF formation energies were performed using density functional theory^45,46^_._ Pseudopotentials and plane waves were used to solve self-consistently the Kohn–Sham equations. The ABINIT code was employed with norm-conserving Trouiller–Martins pseudopotentials to account for nuclei and core electrons^47,48^. The valence comprised 3*d*, 4*s*, 4*p* electrons of gallium and 2*s*, 2*p* electrons of nitrogen. The 3*d* gallium orbitals were explicitly included to improve the accuracy of the calculations^49^. The valence one-electron Kohn–Sham wave functions were expanded in plane waves basis set whose size corresponds to a cut-off energy of 70 Ry. Integrals over the Brillouin zone were evaluated through uniform Monkhorst–Pack grids^50^, which were shifted by (0.5, 0.5, 0.5). A (8 × 4 × 8) grid with 128 symmetry reduced k-points was used for all models. The LDA-type Perdew–Wang functional was employed to describe exchange and correlations effects^51^. Structural optimizations of the unit-cells were performed using the Broyden-Fletcher-Goldfarb-Shanno algorithm^52^, where the convergence criterion of 10^–5^ hartree/bohr was adopted for forces.

## Supplementary information

Supplementary Information.
