# Supplementary material for: The heterogeneous nucleation of threading dislocations on partial dislocations in III-nitride epilayers
Source: Sci Rep. 2020 Oct 15;10:17371. doi: 10.1038/s41598-020-74030-y (PMC7566635; doi:10.1038/s41598-020-74030-y)
Supplement: Supplementary file 1 — Supplementary Information. [file 41598_2020_74030_MOESM1_ESM.docx]

**The heterogeneous nucleation of threading dislocations on partial dislocations in III-nitride epilayers**

J. Smalc-Koziorοwska^1^^[[1]](#footnote-1)^, J. Moneta^1^, P. Chatzopoulou^2^, I. G. Vasileiadis^2^, C. Bazioti^3^,

Ø. Prytz^3^, I. Belabbas^4^, Ph. Komninou^2^, and G. P. Dimitrakopulos^2*^

*^1^Institute of High Pressure Physics, Polish Academy of Sciences, 01-142 Warsaw, Poland*

*^2^Physics Department, Aristotle University of Thessaloniki, 54124 Thessaloniki, Greece*

*^3^Department of Physics, Centre for Materials Science and Nanotechnology, University of Oslo, Blindern, N-0316, Oslo, Norway*

*^4^ Chemistry Department of Abderahmane Mira University, Bejaia, Algeria 06000*

**Supplementary Information**

**Supplementary Table S1.** Visibility criteria for diffraction contrast under two-beam imaging, applied to **a**-type lattice dislocations with line direction **u** = [0001], and to partial dislocations (PDs) with **u** = <1$\bar{1}$00> lines.

|  | **g.b** | **g.**(**bxu**) | **g.b** | **g.**(**bxu**) | **g.b** | **g.**(**bxu**) |
| --- | --- | --- | --- | --- | --- | --- |
| *Burgers vectors of TDs with* **u** = [0001] *line direction* | | | | | | |
| **g** | **b** = ±1/3[2$\bar{1}\bar{1}$0] | | **b** = ±1/3[$\bar{1}$2$\bar{1}$0] | | **b** = ±1/3[$\bar{1}\bar{1}$20] | |
| 1$\bar{1}$00 | ±1 | ±1 | ±1 | ±1 | 0 | ±2 |
| 10$\bar{1}$0 | ±1 | ±1 | 0 | ±2 | ±1 | ±1 |
| 01$\bar{1}$0 | 0 | ±2 | ±1 | ±1 | ±1 | ±1 |
| $\bar{1}\bar{1}$20 | ±1 | ±1 | ±1 | ±1 | ±2 | 0 |
| $\bar{1}$2$\bar{1}$0 | ±1 | ±1 | ±2 | 0 | ±1 | ±1 |
| 2$\bar{1}\bar{1}$0 | ±2 | 0 | ±1 | ±1 | ±1 | ±1 |
| *Burgers vectors of PDs along* **u** = <1$\bar{1}$00> *line directions* | | | | | | |
| **g** | **b** =1/3[1$\bar{1}$00] | | **b** =1/3[10$\bar{1}$0] | | **b** =1/3[01$\bar{1}$0] | |
| $\bar{1}\bar{1}$20 | ±1 | 0 | ±1 | 0 | 0 | 0 |
| $\bar{1}$2$\bar{1}$0 | ±1 | 0 | 0 | 0 | ±1 | 0 |
| 2$\bar{1}\bar{1}$0 | 0 | 0 | ±1 | 0 | ±1 | 0 |
| 1$\bar{1}$00 | ±1/3 | 0 | ±1/3 | 0 | ±2/3 | 0 |
| 10$\bar{1}$0 | ±1/3 | 0 | ±2/3 | 0 | ±1/3 | 0 |
| 01$\bar{1}$0 | ±2/3 | 0 | ±1/3 | 0 | ±1/3 | 0 |

**SUPPLEMENTARY NOTE S1: Topological analysis of the I_3_ BSF domain**

Let us consider initially the reference model of a single I_1_ BSF bounded by a hexagonal Frank-Shockley 1/6[02$\bar{2}$3] PD loop with its sides along the *m*-directions, as shown schematically in Fig. S1(a). Regarding the 1/3[01$\bar{1}$0] Shockley component of the Burgers vector, such a loop would comprise two 0^o^ segments and four 60^o^ ones. There is also the **c**/2 edge (90^ο^) component, i.e. the Frank component of the Burgers vector. The BSF exhibits the RBT **p***_i_* = 1/6[02$\bar{2}$3] [mod(**a***_i_*)], where mod denotes modulo, and **a***_i_* = 1/3<$\bar{1}\bar{1}$20>. If we then overlap a second I_1_ BSF that exhibits the opposite RBT, -**p***_i_,* as illustrated in Fig. S1(b), the net dislocation content is zero and the I_3_ defect is constructed. In the configuration of Fig. S1(b), if the side facets of the hexagonal prism are PSFs of Amelinckx type (see main text) then the domain’s facet junctions do not require defect character. If the PSFs are of Drum type, the I_1_/Drum facet junctions have Burgers vectors **b**_SR-_*_ij_* = **p**_D-_*_j_* – **p***_i_* = 1/6<01$\bar{1}$0>, and there are six geometrically necessary vectors for these screw PDs as illustrated in Fig. S1(c). At the PSF facet junctions we have edge-type stair-rod PDs with **b**_SR_*_-j_* = 1/6<2$\bar{1}\bar{1}$0> as shown in Fig. S1(c).


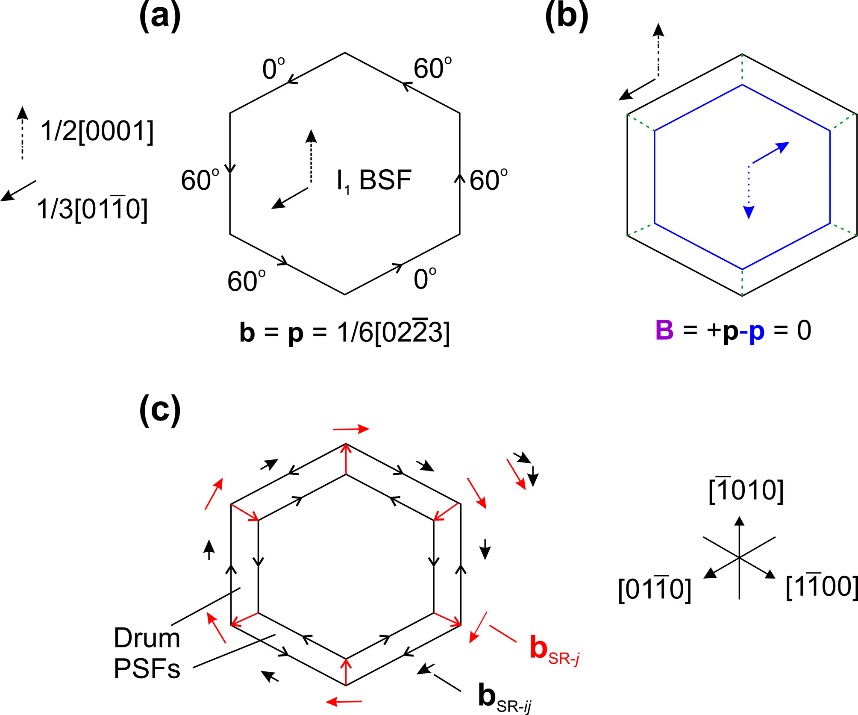


**Figure S1.** (**a**) Schematic along [0001] of a hexagonal I_1_ BSF, bounded by a PD loop with Burgers vector **b** equal to the rigid body translation **p**. The out-of-plane **c**/2 component is denoted by a dashed vector. The sides of the loop are along the *m*-directions, and the sense of the dislocation line is indicated. (**b**) Schematic in perspective view of two overlapped I_1_ BSFs (black and blue) exhibiting opposite **p** vectors, forming an I_3_ prismatic hexagonal domain. (**c**) I_3_ domain comprising Drum PSFs. Burgers vectors and line senses of stair-rod dislocations are indicated. Vectors **b**_SR-_*_ij_* = 1/6<10$\bar{1}$0> and **b**_SR_*_-j_* = 1/6<2$\bar{1}\bar{1}$0> are drawn in black and red respectively.

**SUPPLEMENTARY NOTE S2: Details of the elasticity calculations**

The isotropic elasticity approach described in Ref. [^[[2]](#endnote-1)^] was employed. The elastic energies for the hexagonal loops were calculated from the sum of the self-energies of the six PD segments plus their interaction energies. The self energy of a single segment is given by

$W_{self}=\frac{GD}{8\pi}\left[ \left( \boldsymbol{b}\cdot\hat{\boldsymbol{u}} \right)^{2}\boldsymbol{+}\frac{\left| \boldsymbol{b\times}\hat{\boldsymbol{u}} \right|^{\boldsymbol{2}}}{1-v} \right]\cdot ln\left( \frac{D}{2eR} \right)$ (S1)

where $\hat{\boldsymbol{u}}$ is the unit vector along the dislocation line **u**, *G* and *ν* are the shear modulus and Poisson ratio respectively, *D* is the domain diameter, *R* is the dislocation core radius, and *e* is Euler’s number. The interaction energy for non parallel PD segments is

$W_{ij}=\frac{G}{4\pi}\left\{ \left( \boldsymbol{b}_{\boldsymbol{i}}\boldsymbol{\cdot}{\hat{\boldsymbol{u}}}_{\boldsymbol{i}} \right)\left( \boldsymbol{b}_{\boldsymbol{j}}\boldsymbol{\cdot}{\hat{\boldsymbol{u}}}_{\boldsymbol{j}} \right)-2\left[ \left( \boldsymbol{b}_{\boldsymbol{i}}\boldsymbol{\times}\boldsymbol{b}_{\boldsymbol{j}} \right)\cdot\left( {\hat{\boldsymbol{u}}}_{\boldsymbol{i}}\boldsymbol{\times}{\hat{\boldsymbol{u}}}_{\boldsymbol{j}} \right) \right]\boldsymbol{+}\frac{1}{1-v}[\boldsymbol{b}_{\boldsymbol{i}}\boldsymbol{\cdot}\left( {\hat{\boldsymbol{u}}}_{\boldsymbol{i}}\boldsymbol{\times}\hat{\boldsymbol{z}} \right)\boldsymbol{]}[\boldsymbol{b}_{\boldsymbol{j}}\boldsymbol{\cdot}\left( {\hat{\boldsymbol{u}}}_{\boldsymbol{j}}\boldsymbol{\times}\hat{\boldsymbol{z}} \right)\boldsymbol{]} \right\}I(x_{a},y_{b})$

(S2)

and for parallel PD segments

$W_{ij}=\frac{G}{4\pi}\left( \boldsymbol{b}_{\boldsymbol{i}}\boldsymbol{\cdot}{\hat{\boldsymbol{u}}}_{\boldsymbol{i}} \right)\left( \boldsymbol{b}_{\boldsymbol{j}}\boldsymbol{\cdot}{\hat{\boldsymbol{u}}}_{\boldsymbol{j}} \right)+\frac{G}{4\pi\left( 1-v \right)}\left\{ \left( \boldsymbol{b}_{\boldsymbol{i}}\boldsymbol{\cdot}\hat{\boldsymbol{z}} \right)\left( \boldsymbol{b}_{\boldsymbol{j}}\boldsymbol{\cdot}\hat{\boldsymbol{z}} \right)+\left[ \left( \boldsymbol{b}_{\boldsymbol{i}}\boldsymbol{\times}{\hat{\boldsymbol{u}}}_{\boldsymbol{i}} \right)\boldsymbol{\cdot}\hat{\boldsymbol{z}} \right]\left[ \hat{\boldsymbol{z}}\cdot\left( \boldsymbol{b}_{\boldsymbol{j}}\boldsymbol{\times}{\hat{\boldsymbol{u}}}_{\boldsymbol{j}} \right) \right] \right\}I\left( x_{a},y_{b} \right)$ . (S3)

In Eqs. (S2) and (S3), $\hat{\boldsymbol{z}}$ = [0001], and *I*(*x_a_,y_b_*) is a geometrical factor defined by the position of each segment relative to the other.

Using the above three equations, the resulting total energy, *W_loop_* for the zonal PD loop of Fig. 5(b) is found to be

$W_{loop}=\frac{3GB^{2}D}{8\pi}\frac{\left( 2-\nu\right)}{\left( 1-\nu\right)}\left[ \ln\left( \frac{D}{2R} \right)-0.84 \right]$ . (S4)

In Eq. (S4), the magnitude of the Burgers vector **B** of the dislocation is *B* = 3^-1/2^*a* where *a* = 0.3189 nm is the lattice constant of GaN [^[[3]](#endnote-2)^]. In our calculations, the isotropic elastic constants were obtained using the Voigt-Reuss-Hill approximation [^[[4]](#endnote-3)^] from anisotropic elastic constants that were calculated by density functional theory [^[[5]](#endnote-4)^]. The used values were *G* = 124.1 GPa, and *ν* = 0.2.

For the configuration of Fig. 5(c), the total energy, *W*_s_, of the six 1/3<1$\bar{1}$00> screw PD segments is found to be

$W_{s}=\frac{3GB^{2}D}{4\pi}\left[ \ln\left( \frac{D}{2R} \right)-0.71 \right]$ (S5)

The core radius of the 1/3<1$\bar{1}$00> PDs along *m*-type line directions was tentatively taken equal to *R =* 0.4 nm, approximately equal to that of *m*-line Shockley partials (based on atomistic calculations to be presented elsewhere). We then consider the energy required to nucleate the six 1/3<$\bar{1}\bar{1}$20> lattice TDs. Their nucleation energy can be approximated by their elastic self energy, and the total is given by

$W_{TD}=6\cdot d\cdot\frac{G}{4\pi}\frac{{b_{L}}^{2}}{(1-v)}\ln\left( \frac{d}{R_{L}} \right)$ (S6)

where *d* is the TD segment length. The core radius *R*_L_ = 0.6 nm for a 5/7 core was used [^[[6]](#endnote-5)^].

Let us now consider Fig. 5(d), whereby the energy *W*_s_ is replaced by two terms as summarized in Eq. (2). The first concerns the total energy of six pairs of 1/6<1$\bar{1}$00> stair-rod PDs, given again by Eq. (S5), except that *B* is replaced by *b*_sr_ *=* *B*/2, i.e.

$W_{sr}=\frac{3Gb_{sr}^{2}D}{2\pi}\left[ \ln\left( \frac{D}{2R_{sr}} \right)-0.71 \right]$ (S7)

where we assumed *R*_sr_ ≈ *R*/2. The second is the repulsive interaction between the stair-rod PDs leading to an energy gain given by the term

$W_{int}$ = $3D\int_{2R_{SR}}^{z} \frac{{Gb_{sr}}^{2}}{2\pi}\frac{dz}{z}$ (S8)

where *z* is the domain height (Fig. 5(d)), and the minimum distance between the two stair-rod PDs was taken equal to 2*R*_SR_. There is also the energy gain due to the replacement of the Amelinckx PSFs by Drum ones as detailed in the main text.

**References**

1. Corresponding Authors: [julita@unipress.waw.pl](mailto:julita@unipress.waw.pl); [gdim@auth.gr](mailto:gdim@auth.gr) [↑](#footnote-ref-1)
2. [] Hirth, J., & Lothe, J. *Theory οf Dislocations* 159 (Wiley, 1982). [↑](#endnote-ref-1)
3. [] Morkoç, H. Handbook of nitride semiconductors and devices, 9-11 (Wiley, 2008). [↑](#endnote-ref-2)
4. [] Hill, R. The Elastic Behaviour of a Crystalline Aggregate. *Proc. Phys. Soc. Sect.* A **65**, 349–354, <https://doi.org/10.1088/0370-1298/65/5/307> (1952). [↑](#endnote-ref-3)
5. [] Qin, H., Luan, X., Feng, C., Yang, D. & Zhang, G. Mechanical, thermodynamic and electronic properties of wurtzite and zinc-blende GaN. *Materials* **10**, 1419, [https://doi.org/10.3390/ma10121419](%20https:/doi.org/10.3390/ma10121419%20) (2017). [↑](#endnote-ref-4)
6. [] Kioseoglou, J., Komninou, Ph., & Karakostas, Th. Core models of a‐edge threading dislocations in wurtzite III(Al,Ga,In)‐nitrides. *Phys. Status Solidi A* **206**, 1931-1935, <https://doi.org/10.1002/pssa.200881435> (2009). [↑](#endnote-ref-5)
